# Both Conventional and Interferon Killer Dendritic Cells Have Antigen-Presenting Capacity during Influenza Virus Infection

**DOI:** 10.1371/journal.pone.0007187

**Published:** 2009-09-28

**Authors:** Corine H. GeurtsvanKessel, Ingrid M. Bergen, Femke Muskens, Louis Boon, Henk C. Hoogsteden, Albert D. M. E. Osterhaus, Guus F. Rimmelzwaan, Bart N. Lambrecht

**Affiliations:** 1 Department of Pulmonary Medicine, Erasmus University Medical Centre, Rotterdam, The Netherlands; 2 Department of Virology, Erasmus University Medical Centre, Rotterdam, The Netherlands; 3 Department of Respiratory Diseases, University Hospital Ghent, Ghent, Belgium; 4 Bioceros B. V., Utrecht, The Netherlands; NIAID, United States of America

## Abstract

Natural killer cells are innate effector cells known for their potential to produce interferon-γ and kill tumour and virus-infected cells. Recently, B220^+^CD11c^int^NK1.1^+^ NK cells were found to also have antigen-presenting capacity like dendritic cells (DC), hence their name interferon-producing killer DC (IKDC). Shortly after discovery, it has already been questioned if IKDC really represent a separate subset of NK cells or merely represent a state of activation. Despite similarities with DCs, in vivo evidence that they behave as bona fide APCs is lacking. Here, using a model of influenza infection, we found recruitment of both conventional B220^−^ NK cells and IKDCs to the lung. To study antigen-presenting capacity of NK cell subsets and compare it to cDCs, all cell subsets were sorted from lungs of infected mice and co-cultured ex vivo with antigen specific T cells. Both IKDCs and conventional NK cells as well as cDCs presented virus-encoded antigen to CD8 T cells, whereas only cDCs presented to CD4 T cells. The absence of CD4 responses was predominantly due to a deficiency in MHCII processing, as preprocessed peptide antigen was presented equally well by cDCs and IKDCs. In vivo, the depletion of NK1.1-positive NK cells and IKDCs reduced the expansion of viral nucleoprotein-specific CD8 T cells in the lung and spleen, but did finally not affect viral clearance from the lung. In conclusion, we found evidence for APC function of lung NK cells during influenza infection, but this is a feature not exclusive to the IKDC subset.

## Introduction

Influenza type A is a cytolytic virus that causes acute respiratory infection of which the clinical outcome can vary greatly. The way in which the innate and adaptive immune system initially recognizes and deals with replicating virus could be decisive in determining outcome of infection, as this might heavily influence the kinetics of viral clearance [1], [2], [3]. In immediate response to viral infection, innate defence mechanisms consist of high level production of type I interferons by infected epithelial cells, alveolar macrophages and natural interferon producing cells, as well as recruitment of neutrophils and NK cells [4], [5], [6]. NK cells can kill virus-infected cells without prior antigen stimulation [7], [8], [9] in a process that is controlled by inhibitory and activating receptors, of which the activating natural killer cell receptor (*Ncr1*) gene product is most crucial during influenza infection [9], [10], [11]. How exactly this innate immune response influences or enhances initiation of adaptive immunity is poorly understood.

By expressing a wide array of microbial pattern recognition receptors shared with cells of the innate immune response and at the same time displaying the potential to process and present antigen to naive T cells, lung DCs bridge innate and adaptive immunity [12]. During influenza infection, both conventional (cDC) and plasmacytoid (p)DCs exert different functions, but both are necessary to induce an immune response that clears the virus from the lungs and prevents re-infection [13]. Recently it has been proposed that new member in the mouse DC family might display both innate immune functions of NK cells, as well as the potential to process and present antigen to naïve T cells, the so-called interferon producing killer DC, or IKDC [14], [15]. Phenotypically IKDCs are defined as non-T (CD3^−^), non-B (CD19^−^) cells, with expression of intermediate levels of CD11c, B220, and MHC class II, and high-level expression of NK-specific markers NK1.1 and NKp46 controlled by the *Ncr1*-locus [14], [15], [16]. Functionally, IKDCs have the capacity to kill NK-sensitive target cells without prior activation, and induce proliferation of naïve T cells when pulsed with antigenic peptides. By contrast, conventional NK cells that lack expression of B220 and MHCII have not been identified as antigen presenting cells in the mouse [17]. Although the existence of the IKDC population would make evolutionary sense, it has been controversial whether IKDCs really represent a separate DC lineage or nothing else but activated NK cells, endowed with antigen presenting capacity [18]. The latter view is supported by the fact that human NK cells have long been known to have APC-like activity [19], [20]. The developmental pathways between NK cells and IKDCs also overlap as both rely on the IL-2Rβ, IL-5Rβ and common γ chain (and thus a functional IL-15R complex) and IL-15 for development, whereas cDCs and pDCs develop in the absence of a functional IL-15R [15], [16]. Furthermore there was some doubt whether resting or CpG activated splenic IKDCs could present protein antigen or relevant pathogens (as opposed to pre-processed peptide) to CD8 and CD4 T cells *ex vivo* [21]. Therefore, some authors suggest that IKDCs are functionally and developmentally closer to NK cells than to the two best-known DC family members.

Here, we have studied the antigen presenting capacities of conventional NK cells, IKDCs and cDCs during influenza infection. Subsets of conventional NK and IKDCs were sorted from the lungs of infected mice to study their APC potential, in direct comparison with conventional DCs. We found clear evidence for recruitment of NK subsets to lungs of infected mice and both conventional and IKDC subsets were able to present virus-encoded antigen to CD8 cells, but not CD4 T cells. In support, NK1.1 depletion led to a reduced expansion of virus specific CD8 T cells in the spleen and lung. However, viral clearance was unaffected. These data support an APC function for NK cells, which is not unique to the IKDC subset.

## Results

### Surface phenotype analysis of lung DC subsets and NK cells following influenza virus infection

**Figure 1:**
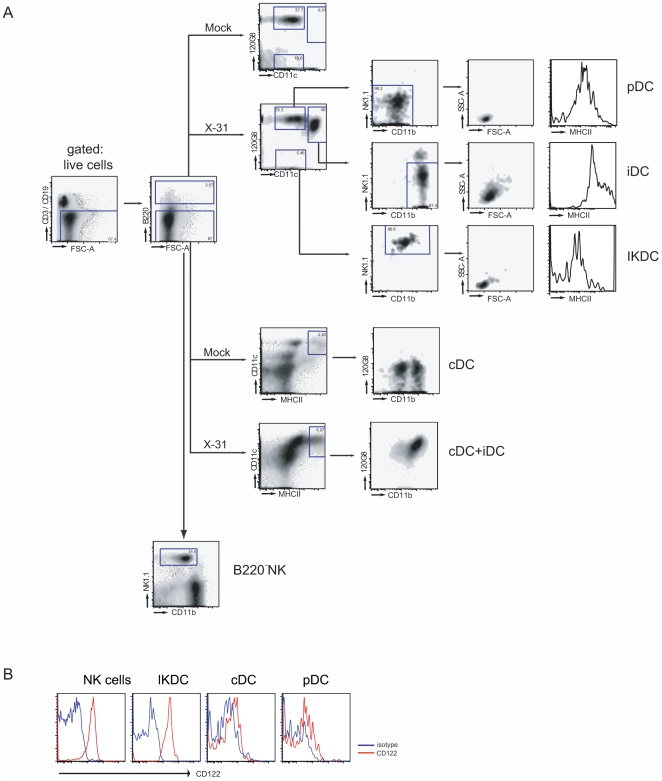
Identification of NK and DC subsets cell in lung tissue. (A) 10-color flowcytometric analysis of lung tissue. A CD3/CD19 negative population was gated from live, low autofluorescent cells in the lung. The B220 high population was further subdivided based on CD11c, 120G8, CD11b and NK1.1. After influenza virus infection, three populations could be clearly demarkated.CD11c^interm^120G8^low^NK1.1^hi^IKDCs, CD11c^interm^120G8^hi^NK1.1^lo^CD11b^lo^ pDCs and CD11c^hi^120G8^hi^CD11b^hi^MHCII^hi^ iDCs. The iDC population was completely absent in steady state condition. In the B220 low population cDCs were gated as MHII^hi^CD11c^hi^ and NK cells as a NK1.1^hi^ population expressing intermediate levels of CD11b. (B) Histograms demonstrating CD122 expression on NK cells, IKDCs and cDCs. The grey histogram represents isotype control, black histogram represents CD122 expression. Representative of 3 experiments.

There is considerable overlap in the level of expression of phenotypical markers that have been used to discriminate putative IKDCs from pDCs, cDCs and NK cells. In an attempt to make a head to head comparison of these various subsets in lungs in steady state conditions (mock infected mice) or during influenza infection, we have employed 10-colour flow cytometry to rigorously define phenotypes and activation status. We first gated on live cells in the lung and subsequently gated out the CD19^+^ B and CD3^+^ T cells (Figure 1A, left plot), while discriminating between B220^+^ and B220^−^ cells. Conventional DCs (cDC) are described as being low for B220, while expressing high levels of CD11c and MHCII. As previously described by others, and us, in steady state conditions (mock) these cells can be further discriminated into a CD11b^+^ and a CD11b^−^ subpopulation lacking expression of 120G8 as depicted in the lower panels of Figure 1A [13], [22]. The CD3^−^CD19^−^B220^−^ population also contained the conventional NK1.1^+^ NK cells. When selecting for CD3^−^CD19^−^B220^+^ cells, we found two populations of CD11c^int^ cells in steady state conditions, which can be further discriminated into pDCs by expression of the pDC marker bone marrow stromal antigen-2 recognized by the moAb 120G8. The 120G8^+^CD11c^int^ population represents NK1.1^−^CD11b^−^ pDCs with low forward and side scatter, whereas the 120G8^−^CD11c^int^ population represents NK1.1^hi^CD11b^int^ IKDCs, also of small size and scatter [14]. In influenza X-31 infected lungs, there was a striking difference in the characteristics of isolated populations found at 4 days post infection. Firstly, within the B220^−^MHCII^hi^CD11c^hi^ cDC, there was a loss of the CD11b^−^ subset, as recently reported, and the remaining CD11b^hi^ DCs co-expressed 120G8 (a pDC marker shown to be induced by interferon production) [23], [13]. Even more strikingly, the B220^+^ cells now also contained a subset of cells that highly expressed CD11c (like cDCs) as well as 120G8 (like pDCs). Further analysis of this population revealed that it expressed very high levels of CD11b and intermediate levels of NK1.1, although a subset of these cells was as high in expression of NK1.1 as IKDCs and NK cells. These cells were however larger and expressed very high levels of MHCII, most likely resembling inflammatory DCs, recently surged from monocytes [24], [25]. Figure 1B demonstrates that the expression of the IL-2-IL-15 receptor β chain (CD122) was only expressed on NK cells and IKDCs, but not on pDCs or cDCs, as previously described [15]. In addition, IKDCs in the lungs expressed NKG2D and PDL1 (also known as B7H-1)(data not shown).

### During influenza infection, both lung IKDCs and B220^−^ NK cells lyse NK-sensitive targets

**Figure 2:**
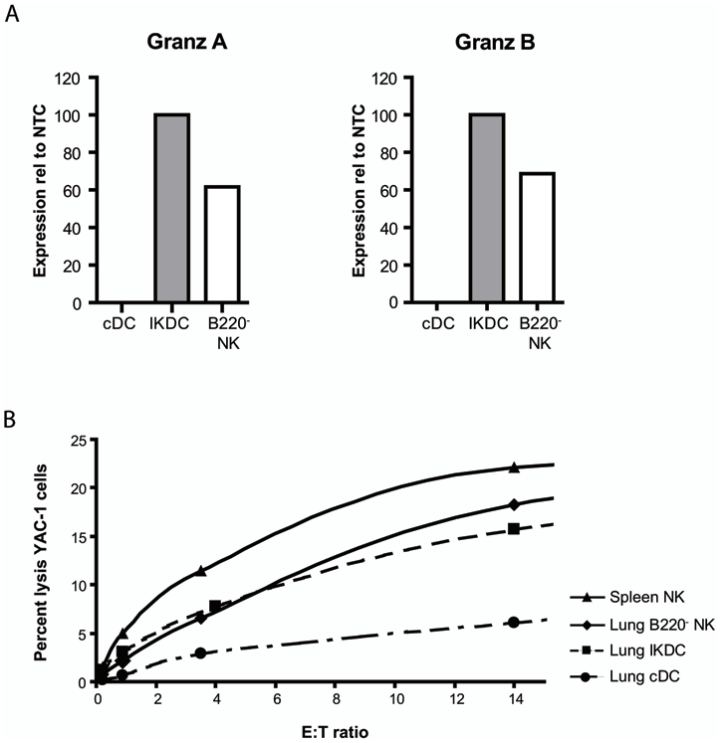
IKDCs and B220^−^ NK cells induce cell lysis. (A) Expression of Granzyme A and B on sorted cell subsets at 4 dpi. Bars represent the values of expression relative to NTC. (B) Percentage YAC-1 cell target lysis after co-culture with different effector DC and NK cell subsets. IKDCs, B220^−^ NK cells and DCs were sorted from lung tissue at 4 dpi following gating strategies depicted in Figure 1 and compared with enriched NK cells from spleen tissue. Cells were incubated with CFSE-labeled NK-sensitive YAC-1 target cells. Lysis at each effector:target (E:T) ratio was determined as described in materials and methods. Representative of 2 experiments.

The main function of NK cells is known to be cell lysis without prior activation in a process requiring granzyme A and B. We compared expression of these molecules by qPCR on lung derived NK cell subsets and cDCs, and found higher expression levels of both Granzym A and B on IKDCs than on regular B220^−^ NK cells. On cDCs no expression was detected (Figure 2A). Next, the functional capacity to lyse NK-sensitive YAC target cells was studied by using a CFSE-labeled YAC assay as described previously [26]. In line with granzyme expression and expression of activating NK receptors, both IKDCs and B220^−^ NK cells sorted from infected lung tissue efficiently lysed YAC-1 target cells (Figure 2B), to a degree that is seen when using splenic NK cells as effector cells, supporting the idea that lung CD3^−^CD19^−^B220^+^CD11c^int^NK1.1^+^ cells also functionally behave like IKDCs [15], [21].

### Kinetics and maturation state of IKDCs, B220^−^NK cells, and cDCs in lung following influenza virus infection

**Figure 3:**
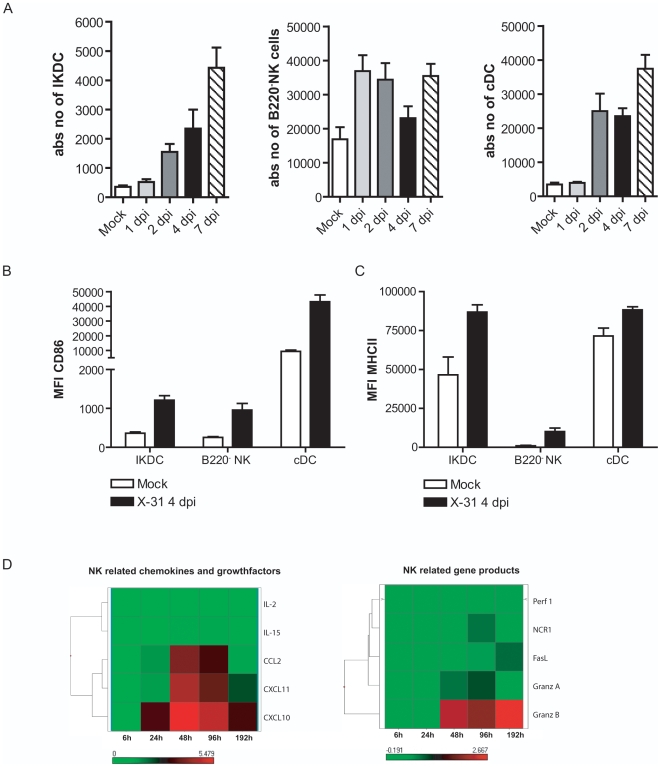
Kinetics of increase in IKDCs, B220^−^NK cells, and cDCs in lung tissue after influenza virus infection. (A) Cell populations were gated as demonstrated in Figure 1. Histograms demonstrate absolute numbers of IKDCs, B220^−^NK cells and cDCs at different time points after infection. Bars represent mean values +/− SEM of at least 5 mice per group. CD86 (B) and MHCII (C) expression on cell populations at 4 dpi expressed as mean MFI +/− SEM, derived from at least five mice per group. Similar results were obtained from three separate experiments (D) Gene expression analysis using Aff ymetrix GeneChips. Top heat map shows NK related chemokines and growth factors. Lower map shows NK related gene products.

As influenza X-31 infection is a mild infection localized in the respiratory tract, we studied the effect of infection on the number and maturation status of IKDCs in the respiratory tract (lung, bronchoalveolar compartment (BALf), mediastinal LN (MLN) and the spleen), using the multi-parameter gating strategy as above. A kinetic analysis at various days post infection (dpi) revealed that IKDCs, B220^−^NK cells and cDCs accumulated in the lungs with different kinetics and to different extents; IKDCs and cDCs peaking at 7dpi whereas NK cells were increased from day 1 to 7 post infection compared with mock infected mice (data shown for digested lung tissue). For clarity reasons we lumped CD11b^+^ and CD11b^−^ cDCs together, but we have recently provided detail on this [13]. We next defined the co-stimulatory molecule expression on these various populations at 4 dpi. Whereas we could find an increased number of IKDCs in all compartments (Figure 3A depicts only the digested lung samples), the increase in expression of the maturation marker CD86 (Figure 3B) and expression of MHC class II (Figure 3C) molecules was restricted to the site of primary infection being lung and BALf (figures depict lung tissue) and occurred on both IKDCs, B220^−^NK cells and cDCs, albeit to different degrees. Notably there were no signs of cDC, NK or IKDC activation in spleen or MLN (data not shown).

An increase in IKDCs could be due to increased chemoattraction of IKDCs to the lung, in a process that might be controlled by CXCR3 and/or CCR2 [27]. We therefore examined expression levels of the CXCR3 ligands CXCL10 (IP-10), CXCL11 (ITAC), as well as the CCR2 ligands CCL2 (MCP-1) on a genome-wide expression micro array performed on whole lung homogenate from mock-infected or X-31 infected mice at various hours post infection (hpi) (Figure 3D, full microarray data to be published elsewhere). Analysis revealed that these chemokines were clearly upregulated with similar kinetics as gene products associated with NK and/or IKDC function such as *Ncr1*, *granzyme A and B*, *klrk1*, *Nkg7*. Alternatively, IKDC accumulation could be due to increased proliferation or survival inside the lung, possibly due to the presence of homeostatic cytokines of the IL-2 family that share the γc chain for signaling (IL-2, IL-15, IL-21). Based on array data, there was induction at 2 dpi in the lung of IL-15, but not IL-2 or IL-21 in X-31 infected mice, whereas expression levels of IL-15rα and IL-2rβ were unaltered (data not shown).

### Antigen presenting capacity of sorted IKDCs, B220^−^NK cells, and cDCs from infected lungs

**Figure 4:**
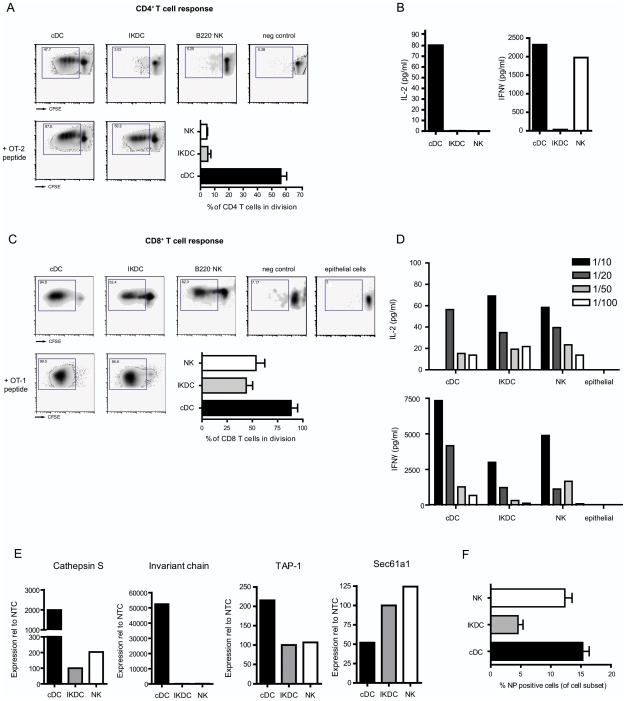
Antigen-presenting capacity of lung cDCs and NK cell subsets. Lung populations were sorted following the gating strategies in Figure 1. (A) CFSE-labelled OT-2 (CD4^+^) – T cell proliferation 4 days after co-culture with cDCs, IKDCs and B220^−^ NK cells sorted from lung tissue of mice infected with PR-8 containing the OVA-CD4^+^ epitope. Negative control sample indicates co-culture of OTII cells with cDCs derived from a mouse infected with a MHCI-OVA epitope containing virus. Histogram represents average % of cells divided, from 3 seperately performed experiments. Lower left plots indicate T cell proliferation after addition of OT-2 peptide to the co-cultures. (B) Histogram represent IL-2 and IFN-γ levels in supernatant of OTII co-cultures (C) Panel shows the same data for CFSE-labelled OT-1 (CD8^+^) - T cell proliferation 4 days after co-culture with cDCs, IKDCs, B220^−^ NK cells and CD45-negative epithelial cells sorted from lung tissue of mice infected with WSN containing the OVA-CD8^+^ epitope. Negative controls in this setting are co-culture of OTI cells with cDCs derived from a mouse infected with a MHCII-OVA epitope containing virus. Numbers in top left corners of all plots represent the percentage of cells recruited into cell division and the histogram shows average % cell divisions of 3 separate experiments. (D) Histograms represent IL-2 and IFN-γ levels in supernatant of OTI co-cultures with different ratios of DCs versus OTI T cells. (E) Expression of TAP-1, Sec61a1, Cathepsin S and invariant chain on sorted cell subsets at 4 dpi. Bars represent the values of expression relative to NTC. (D) Percentage of cells that have nucleoprotein (NP) positivity, measured by flowcytometry, and indicating replicative infection. Bars represent an average of at least 5 mice/group +/− SEM.

After observing a clear increase in absolute number and maturation state (MHCII and CD86) of IKDCs in lung tissue at 4 dpi, we decided to sort IKDCs from infected mice and perform a head to head comparison with their two closest family members, cDCs and B220^−^ NK cells for the capacity to present to CD8 or CD4 T cells. To study the antigen presenting capacity of the different cell subsets we infected mice either with an influenza virus containing the OVA MHCI (SIINFEKL) or MHCII OVA_323–339_ epitope. At 4 dpi DC and NK cell subsets were sorted with a purity exceeding 98% from the lung tissue and put in co-culture with either CFSE-labeled OVA specific CD8^+^ T cells (OT-1) or CD4^+^ T cells (OT-2), taken from the respective TCR transgenic animals. As a negative control, co-cultures were set up with influenza virus infected cDCs in which the opposite OVA epitope was expressed (for example: a virus containing the MHCII OVA epitope for presentation to OT-1 cells). To test if any lung cell exposed to the innate cytokine response would be able to induce proliferation of OT-1 T cells, additional cocultures were set up with CD45^−^ lung epitelial cells as APCs. Four days later, CFSE dilution of T cells was measured by flowcytometry, and expressed as the % of cells that had divided at least once. As shown in Figure 4A, cDCs induced strong CD4 T cell proliferation *ex vivo* following influenza infection (48% recruited into cell division), whereas IKDCs only induced the proliferation in 3% and B220^−^NK cells in 5% of co-cultured CD4 T cells. In the supernatant of the co-culture we determined levels of IL-2 and IFN-γ (Figure 4B) and according to the T cell division we found high levels of IL-2 in the co-culture with cDCs. High levels of IFN-γ were induced in the co-culture with cDC and B220^−^ NK cells but not with IKDCs. Next, we studied the capacity to induce CD8 T cell proliferation (Figure 4C). Conventional DCs induced the proliferation of up to 95% of co-cultured CD8^+^ Ag specific T cells. In contrast to the lack of induction of CD4 responses, IKDCs presented influenza derived OVA epitopes to CD8^+^ T cells (53% recuited into cell division), however to a degree similar to B220^−^NK cells (62% recuited into cell division). Lung epithelial cells did not induce any T cell divisions. Cytokine profiles in Figure 4D supported these data; high levels of both IL-2 and IFN-γ were measured in the cDC co-culture, whereas similar (yet lower) levels were found in cultures with IKDCs and NK cells, and epithelial cells failed to induce IL-2 or IFN-γ. The cytokine production in supernatant showed a clear dose dependency in all APC populations, which were put into co-culture with several concentrations of OT-1 T cells. These data correlated with intra-cellular staining of IFN-γ (data not shown) The absence of CD4 T cell response induction in the face of clear CD8 activation could signify a defect in processing of viral antigen for MHCII pathway or an absence of co-stimulatory molecules on NK subsets. To address the maximum amount of proliferation possible in co-cultures of purified lung APCs and purified T cells, we added pre-processed MHCII-restricted OVA_323–332_ peptide to the co-culture of IKDCs and OT-II cells, which induced efficient proliferation in up to 50% of CD4 cells, similar to the degree of division seen in T cells stimulated with cDCs (67%). Likewise, addition of MHCI-restricted OVA peptide led to identical T cell proliferation induced by IKDCs and cDCs. It has been proposed that specializations of DCs to either cross-present to CD8 cells or to CD4 cells is a function of particular DC subsets caused by the expression of specific enzymes involved in antigen processing in particular DC subsets, or differential expression of antigen-uptake receptors [28], [29]. To study antigen-processing capacity of the various isolated NK-like cell populations, quantitative PCRs were performed on the sorted cell subsets (Figure 4E). Class II processing and loading molecules (cathepsin S and invariant chain) were expressed in high levels by cDCs and to a much lesser extent in IKDCs or B220^−^NK cells. MHCI pathway molecules Sec61a1 (an endoplasmatic reticulum membrane protein translocator involved in crosspresentation to CD8 T cells) and TAP-1 (ATP-binding cassette transporter associated with MHCI loading) were expressed on cDCs and on both IKDC and B220^−^NK cells. Differences in APC capacity might also reflect different degrees by which these cells were infected by influenza virus. To address this point, we studied the degree of infection by staining for nucleoprotein (NP) in the separate cell subsets at 4 dpi [13]. Whereas 15% of cDCs and B220^−^ NK cell isolated from the lung were infected, we found that only 5% of IKDCs were NP positive (Figure 4F).

### Depletion of NK1.1 positive cells during influenza virus infection reduces the innate and adaptive immune response to influenza

**Figure 5:**
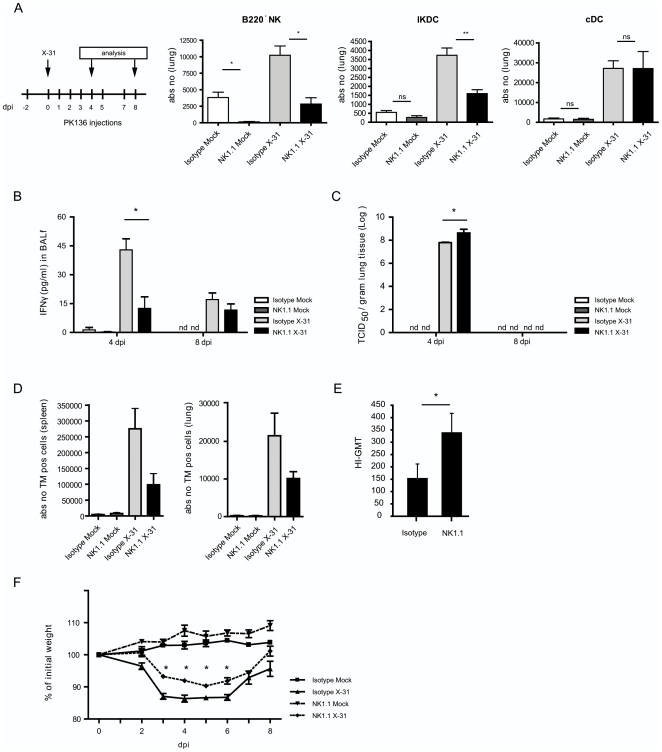
Depletion of NK1.1^+^ cells during in vivo influenza virus infection affects infection parameters. (A) 2 days after injection of PK136 (NK1.1 depleting antibody), mice were infected with X31 virus. Two days later, depletion of NK subsets and cDCs was checked in lung tissue by using the gating strategy in Figure 1. (B) IFN-γ was measured in BALf of infected mice versus mock-infected mice with or without NK1.1^+^ cell depletion. Bars represent mean values of IFN-γ +/− SEM. At least 5 mice per group were used, * p<0,05. (C) Viral titers were measured in lung tissue of at least 5 mice per group and expressed as mean TCID_50_ +/− SEM. * p<0,05. (D) Absolute numbers of TM specific CD8^+^ T cells were measured in spleen and lung tissue at 8 dpi and represented as mean values +/− SEM, of at least 5 mice/group. (E) Hemagluttinin specific antibodies in serum were measured at 8 dpi and depicted as geometric mean titer +/− SE. * p<0,05. (F) Weight loss of mice during infection depicted as % of initial weight. Values indicate mean of at least 5 mice per group +/− SEM.*p<0,05. The demonstrated data are representative of 4 independently performed experiments.

The described experiments suggested that IKDCs and B220^−^ NK cells had APC potential for CD8 cells *ex vivo*. To study the role of NK1.1^+^ cells during infection *in vivo* we injected the NK1.1-specific depleting antibody PK136 before and throughout influenza infection or mock infection [30]. Both in mock infected and infected mice, intra-peritoneal (i.p.) injection of the antibody efficiently depleted NKG2D^+^ B220^−^NK cells and IKDCs from the lung tissue (Figure 5A) when compared to injection with isotype Ig diluted in PBS, although depletion was more absolute in mock infected mice. On the contrast, cDC numbers were unaffected by treatment with NK1.1. Previously an enhancing effect of NK cells on the state of maturation and antigen-presenting capacity of cDCs has been suggested [31], [32], [33]. To exclude any confounding effects of NK1.1 depletion on cDC functions, we additionally compared the antigen presenting capacity of sorted cDCs after infection with influenza virus containing the OVA MHCI epitope, in mice treated or not with PK136. At 4 dpi cDCs were sorted from both groups and expressed similar levels of co-stimulatory molecules (CD86) and MHCII and induced the same amount of T cell proliferation (Figure S1). We next addressed the impact of NK1.1 depletion on induction of antiviral innate and adaptive immunity to a mild X-31 pulmonary influenza infection. During the early innate response to influenza, IFN-γ is found in high levels at the site of infection (lung tissue and BAL fluid) [13] and NK cells are the most prominent source of this cytokine [6], [11]. Not surprisingly, we found a decrease of IFN-γ at 4 dpi in mice depleted of NK1.1 cells. At 8 dpi this mild influenza X-31 infection has been cleared from the lungs and therefore IFN-γ levels start decreasing at this time point (Figure 5B). Viral titers in mice lacking NK1.1 cells were increased 5 fold at 4 dpi compared with mice treated with isotype control antibody. Nevertheless virus was efficiently cleared at 8 dpi (Figure 5C). Most importantly, to address the contribution of NK1.1 positive cells on induction of adaptive CTL immunity, we measured the percentage of virus-specific CD8 T cells at day 8 post infection by using a PE labeled H-2D^b^ tetramer with the NP_366–374_ epitope ASNENMETM [13]. At day 8 we found decreased numbers of tetramer (TM) positive cells in both spleen and lung tissue (Figure 5D). In addition to this we determined the hemagluttinin specific antibodies in serum at 8 dpi (Figure 5E) and found increased levels following NK1.1 depletion, supporting the higher viral titers. Both at earlier and later time points, similar conclusions on tetramer positive cell induction and humoral immune responses were reached (Figure S2). Surprisingly, NK1.1 depleted mice did not appear to have much more severe systemic morbidity as their weight loss did not exceed 10% of initial weight, in contrast with isotype treated mice, which lost significantly more weight during the peak of infection (Figure 5F). As a final support for an antigen presenting capacity of NK cells *in vivo*, we also found colocalization of naïve OVA-specific CD8 T cells with NK cells in the draining nodes 48 h after infection with influenza-OVA virus but to a much lower extent with wild type influenza virus (Figure S3).

## Discussion

NK cells are part of the innate immune system and among the first immune effector cells to arrive at inflammation sites [5], [11]. Here, we found rapid accumulation of cells with an IKDC phenotype in infected lungs. The reason for this could be enhanced recruitment of IKDCs to the lungs, or enhanced proliferation and/or survival of IKDCs in the infected lung. Although little is known about chemokines and their receptors that govern directed migration of IKDCs, CCR2 and CXCR3 are likely candidates based on a recent study [27] and similarities with NK cell migration [34]. Accordingly, we found an early yet persistent upregulation of the ligands CXCL10 and CXCL11 as well as CCR2 and CCL2 in virus-infected lungs. We also found increased expression of IL-15 in the lungs, a critical cytokine involved in IKDC development, expansion and survival in steady state [16], [21], [23] and inflammatory conditions [27].

Another likely explanation for the increased number of NK-like cells in the lungs following infection could be that cells are interconverting by differentiation, thereby upregulating markers on their surface. We do not exclude that IKDCs could be a highly differentiated NK cell but decided to study both NK cell subsets separately and compare them with cDCs in lung tissue.

Although the predominant effector function of NK cells is the direct attack of virus-infected cells, as illustrated here by an increase in early viral titer in the lungs of NK1.1 depleted mice, increasing evidence suggests that they shape the quality and quantity of the ensuing adaptive immune response by either stimulating or suppressing the function of bona fide APCs like DCs [31], [32], [33], [34], [35], [36]. NK cells have extensive trafficking capabilities and are found within non-lymphoid and lymphoid tissues, sometimes in close proximity to naïve T cells, as also shown in this study [34], [37]. *In vitro* studies revealed that activated human NK cells and NK cell clones can process soluble antigen, up-regulate MHCII and co-stimulatory molecules, and thus stimulate naïve CD4 and CD8 T cell responses [19], [20]. The functional relevance of this has been questioned *in vivo*, as mouse NK cells were classically not found to have direct APC function [17], [18]. Recently, it was proposed that IKDCs found in central lymphoid organs and tumours are the *in vivo* mouse correlates of human NK cells that display DC-like properties, such as expression of CD86, MHCII and potential to present pre-processed peptides to T cells [14], [15], [17]. Although in the original description, these cells were proposed to be a separate and new subset of NK-like DCs, subsequently three different groups refuted this idea and proposed that IKDCs are nothing else but DC-like NK cells [16], [21], [23].

Regardless of these ‘family affairs’ between NK cells and DCs, the argument that NK cells might be endowed with APC potential made us embark on a detailed study in the mouse in which we evaluated the precise contribution of NK cells to induction of adaptive immunity during influenza infection of the lung. We repeated an experiment previously reported by Kos and Engleman in which depletion of NK1.1^+^ cells was shown to hamper the induction of virus-specific CD8 CTLs [30]. This experiment had been performed in an era when it was impossible to identify virus-specific CD8 T cells with MHCI-peptide tetramers and multi-colour flow cytometry was not so advanced as to discriminate and sort multiple subsets of NK cells and DCs simultaneously. In our hands, injection of an NK1.1 depleting antibody effectively depleted the NKG2D^+^ conventional B220^−^NK cells, as well as IKDCs, which led to a reduction of tetramer-positive NP_366–374_-specific CD8 T cells upon viral infection, as measured at several time points post infection. Although our data on reduced CTL frequency support the early work of Kos et al, the interpretation has been that NK cells promote the accessory function of DCs via secretion of IFN-γ or direct cell-cell contact [30], [31]. However several other explanations are plausible. By studying the expression of MHCII, CD86 and the potential of cDCs to present viral derived antigen to CD8 T cells, we found little evidence for altered DC accessory function when NK1.1-positive cells were depleted during influenza, lending little support to the former theory. Alternatively, NK1.1 Ab treatment might have eliminated CD8 CTLs directly, as NK1.1 is expressed by some 10% of virus specific CD8 CTLs ([38] and our own unpublished data). We do not believe that direct killing of CTLs by NK1.1 antibody was responsible for the observed effect because as many as 50% of tetramer-positive CD8 cells were depleted. CD3-positive NK T cells also express the NK1.1 marker, yet these cells were recently shown not to play a predominant role for antiviral immunity during influenza infection [39]. Our data on the antigen presenting capacities of NK cell subsets *ex vivo*, as well as the colocalization of NK cells and naïve CD8 T cells in the central lymphoid organs at early time points post infection favour the theory that CD8 T cell responses were partially reduced because of the depletion of B220^−^NK cells and IKDCs, as direct actors in the process of antigen presentation to naive antigen-specific CD8 T cells.

As recently suggested by others, we also found that during influenza infection the APC functions of NK cells are present within a subset of lung CD3^−^CD19^−^NK1.1^+^ B220^+^CD11c^+^ cells that also express killing activity for classical NK targets [14], [15]. However, this APC feature was not unique to IKDCs and similarly present in B220^−^ conventional NK cells. Both subsets upregulated the expression of CD86 upon influenza infection and presented to CD8 T cells. This could be a reflection of direct Ag presentation in case of direct infection of NK subsets by influenza virus or cross-presentation of virally infected lung epithelial cells. At least for IKDCs we favour the latter explanation as we did not find real evidence of direct infection of IKDCs by influenza (low levels of viral nucleoprotein staining compared with cDCs [13], [21]), and as IKDCs highly expressed members of the ER retro translocation machinery Sec61a1 (involved in cross-presentation) and cathepsin S, also recently implicated to play a role in crosspriming [28], [29]. How exactly IKDCs acquire antigen for cross-presentation however is unclear at present as we did not address their capacity to phagocytose virus infected cells, nor the causal relationship between killing of virus infected cells and subsequent processing of antigen derived from killed target cells. The division that was induced *ex vivo* in CD8 T cells by IKDCs was not accompanied by vigorous IFN-γ production, in contrast to T cells stimulated with cDCs under identical conditions.

One factor that discriminates lung NK cell subsets is the higher level of expression of MHCII on IKDCs compared with B220^−^ NK cells in infected lung samples. It was therefore striking to see that IKDCs hardly presented influenza derived antigen to specific CD4 T cells, whereas cDCs readily did under these conditions. The most obvious explanation would be the much higher level of co-stimulatory molecules and MHC molecules on cDCs, and expression of the full enzymatic machinery to process antigen for the MHCII pathway in cDCs. Supporting this idea, we did find that addition of pre-processed MHCII peptide on IKDCs led to T cell divisions, albeit at reduced vigour compared with similarly pulsed cDCs. The apparent lack of mRNA expression of invariant chain by B220^−^NK cells and IKDCs, compared with cDCs explains the incapacity to process for MHCII, as invariant chain is crucial for proper intracellular routing and loading of MHCII molecules by endosomal cargo [28], [29], [40]. Strikingly, the coculture of B220^−^ NK cells and naïve CD4 T cells, did lead to a good production of IFN-γ. The fact that this was not accompanied by CD4 T cell division, makes it likely that this IFN-γ response was NK-derived. The idea that IKDCs have MHC processing capacity has been questioned by other studies showing a very weak potential of these cells to present antigen to CD4 T cells *ex vivo* following expansion in Gleevec/IL-2 and maturation by IL-15 [27], or following exposure *in vitro* to soluble protein antigens or pathogens [21]. It is possible that IL-15 present in the lungs of influenza infected mice (as detected from our whole lung mRNA array data) shuts down part of the APC function of IKDCs for CD4 T cells, as recently demonstrated *in vitro* [27].

At first sight our data on the APC capabilities of IKDCs are at odds with recent papers studying this subject. *In vitro* work in the human immune system illustrated how activated NK cells expressing MHCII and co-stimulatory molecules could process influenza hemaglutinin (HA) protein and virus infected targets for presentation to HA-specific CD8^+^ and CD4^+^ T cell clones [20]. There could be major differences between *in vitro* activated human NK cells and *ex vivo* lung derived murine NK cells that have been part of an innate immune response to live influenza infection. In one of the papers refuting the idea that IKDCs represent a subset of DCs it was shown that splenic IKDCs were unable to present viral antigens to influenza specific T cells following *in vitro* influenza infection, even after CpG stimulation [21]. This could reflect the need for a cell population or cytokine response *in vivo* that licences NK cell subsets to become APCs at the site of infection. Clearly, finding such a stimulus could be very instructive in finding ways to improve mucosal vaccines.

In conclusion we have demonstrated that influenza virus infecting the lung tissue seriously affects the phenotypical appearance of cells, which makes multi-color flowcytometry necessary to distinguish multiple cell subsets. We found *in vivo* and *ex vivo* evidence for antigen presenting capacities within IKDCs and conventional NK cells during influenza infection. All together, clearance of influenza virus from the lungs is therefore yet another example of the delicate crosstalk between innate and adaptive immune response that emerged through evolution to safeguard the host from infectious threats while avoiding immune pathology to the host.

## Materials and Methods

### Influenza virus infection

C57BL/6 mice (6–8 weeks) were purchased from Harlan (Zeist, The Netherlands). Influenza virus X-31 (MRC, Cambridge, England) was inoculated in the allantoic cavity of embryonated chicken eggs. Infectious virus titers were determined in Madin-Darby Canine Kidney (MDCK) cells. Lungs were homogenized and ten-fold serial dilutions of these samples were used in eight-fold to determine the virus titers in MDCK cells as described previously [41]. All experiments were approved by an independent animal ethics committee at Erasmus MC Rotterdam, the Netherlands.

### NK1.1 cell depletion

Naive C57BL/6 mice were treated i.p. with 200 µg of anti-NK1.1 mAb (PK136) on day −2 followed by infection with influenza X-31 at day 0. Subsequent i.p. injections of 100 µg anti-NK1.1. were given at day 0, 1, 2, 3, 4, 5 and 7 post infection. As a control mice were treated with isotype IgG from mouse serum (Sigma-Aldrich).

### Flow cytometry and cell sorting

For detection and phenotyping of DC subsets in organs, cell suspensions of lung, MLN and spleen were prepared as described previously[42]. Cells were subsequently stained with moAbs directed against 120G8 FITC (kindly provided by C.Asselin-Paturel), CD11b PE, MHCII PECy5, B220 PECy7, NKG2D PECy7, CD122 Pacific Orange (eBioscience), CD11c PETxR (Caltag), NK1.1 APC, CD3 APC Cy7, CD19 APC Cy7 (BD Biosciences), and a live/dead marker (DAPI) in Violet. Acquisition of 9–10 color samples was done on a LSRII cytometer. Cell sorting was performed on a FacsARIA cytometer and after cell sorting purity was checked and sorted cells were counted in a Burker cytometer using Trypan Blue. For measurement or viral nucleoprotein (NP) in cells FITC labeld anti-NP antibody (DakoCytomation) was used. Virus-specific CTL were detected by tetramer-staining with following antibodies: CD3e-PerCP, CD8b.2-FITC (PharMingen, San Diego, United States), ToPro 3-APC (Molecular Probes,Eugene, United States) and PE labeled H-2D^b^ tetramer with the NP_366–374_ epitope ASNENMETM (Sanquin Research, Amsterdam, The Netherlands). Final analysis and graphical output were performed using FlowJo software (Treestar, Costa Mesa, CA).

### Flow cytometry–based cytotoxicity assay

Effector cells from infected lungs were sorted on a FacsARIA cytometer and as a positive control spleen NK cells (isolated from infected spleens of the same mice of which lungs had been obtained) were enriched by MACS cell sorting with DX-5 antibodies according to manufacturer's protocol (Miltenyi Biotec GmbH). All effector cells were labeled with CFSE [43] and plated in serial dilution. Target cells (YAC-1) were harvested in exponential growth phase, washed and plated 2000 target cells/well. As a positive control 2% triton was added to a number of wells containing only YAC-1 cells. Cells were centrifuged for 1 minute at 335 g to facilitate cell-cell contact, and incubated for 4 hours at 37°C. At the end of the incubation time samples were put in an ice water bath and Micro Beads were added to allow a constant number of measuring on the LSRII Flowcytometer (BD Bioscience, USA). Prior to measuring, DAPI was added for labeling of dead cells. All samples were analysed using FlowJo software (Treestar, Costa Mesa, CA) as described previously [26].

### Antigen presentation assays

For antigen presentation assays of lung DC subsets, mice were infected with WSN influenza virus encoding OVA_257–264_ K^b^ restricted MHC I epitope in the neuraminidase [44] and PR-8 influenza virus encoding OVA_323–339_ MHC II epitope in hemagglutinin of the virus [45]. The OVA viruses were kindly provided by Dr. R.Webby (St. Jude Children's Hospital, Memphis, Tennessee, USA). OT-1 and OT-2 transgenic T cells were isolated from spleens and LN of respective mice, enriched by MACS cell sorting with anti-CD8 or –CD4 antibodies according to manufacturer's protocol (Miltenyi Biotec GmbH) and labeled with CFSE[43]. Sorted DC subsets were counted and alive cells were co-cultured with T cells at 1/10 ratio for 4 days. T cell divisions were measured by flow cytometry and supernatants were collected and stored at −20°C until ELISA for IFN-γ and IL-2 (BD Biosciences) was performed in 96 wells ELISA plates (Greiner Bio-One).

### Affymetrix GeneChip hybridization and analysis

Mice were infected with 10^5^ influenza virus X-31 at day 0. At several time points after infection (6, 24, 48, 96, 192 hours) mice were sacrificed, lungs were collected in RNAeasy and stored at −80°C. Total lung RNA from influenza virus infected lungs was isolated using an RNeasy kit (Qiagen). RNA was biotin labeled and hybridized to mouse micro arrays (Affymetrix Mouse 430.2).

### Real-time quantitative RT–PCR

Quantative RT-PCR for TAP-1, Sec61a1, Cathepsin S, invariant chain, Granzym A and Granzym B were performed on RNA from sorted DC subsets and NK cells. Frozen cell pellets were homogenized, RNA was isolated with RNAqueous micro kit (Ambion) and treated with DNaseI, according to the manufacturer's protocol. RNA (100 ng) was reverse transcribed using SuperscriptII (Invitrogen) and random hexamers (Amersham Biosciences) for 50 min at 42°C. Quantitative PCR was performed with Taqman Universal PCR Mastermix (Applied Biosystems) and preformulated primers and probe mixes (‘Assay on Demand’, Applied Biosystems). PCR conditions were 2 min at 50°C, 10 min at 95°C, followed by 40 cycles of 15 s at 95°C and 60°C for 1 min using an ABI PRISM 7300 (Applied Biosystems). PCR amplification of the housekeeping gene encoding ubiquitin C was performed during each run for each sample to allow normalization between samples.

### Statistical analysis

All experiments were performed using 5–10 animals per group. The difference between groups was calculated using the Mann-Whitney U test for unpaired data (GraphPad Prism version 4.0; GraphPad, San Diego, CA). Differences were considered significant when p<0,05.

## Supporting Information

Figure S1 Effect of NK1.1 depletion of APC capacities of lung cDCs. (A) Expression of CD86 on cDCs taken at 4dpi from the lungs of influenza infected mice, that were treated with depleting NK1.1 antibody or isotype control. (B) Antigen presenting capacity of cDCs sorted from lung tissue at 4 days after infection with WSN containing an OVA-CD8+ epitope. Sorted cells were co-cultured for 4 days with CFSE-labelled OVA specific CD8 T cells (OT1). A comparison was made between cDCs sorted from NK depleted mice (NK1.1-) versus non-depleted mice, which received an isotype control injection (PBS). Numbers in top left corners represent the percentage of cells recruited into cell division.
(4.77 MB TIF)

Figure S2 Depletion of NK1.1+ cells during in vivo influenza virus infection affects infection parameters at various time points following infection. Mice were treated with depleting NK1.1 antibody or isotype control during influenza virus infection. (A) Viral titers were measured in lung tissue at day 5, 7, and 13 post infection. (B) Left plot shows TM specific CD8+ T cells measured in MLN at day 5, 7, and 13 pos infection. Right plot demonstrates IFNγ producing CD8+ T cells in MLN, measured by intra-cellular staining. (C) % of CD4 T cells in MLN following infection. (D) Hemagluttinin specific antibodies in serum were measured at 8 dpi and depicted as geometric mean titer±SE. In all experiments at least 5 mice per group were used and values are expressed as mean±SEM. * p<0,05.
(18.94 MB TIF)

Figure S3 Colocalization of NK cells and naïve CD8 T cells in MLN at early time points post infection. Adoptive transfer of naïve Ly5.2 OTI CD8+ cells (reactive to OVA class I epitope) was performed to Ly5.1 recipients, allowing to detect OVA specific CD8 T cells using a Ly5.2 antibody. 1 day later mice were immunized with influenza virus containing the OVA class I epitope (Flu (OVA insert)) or with wild type influenza virus (Flu). At 2 days post infection MLN were stained for NK cells with an antibody against NK1.1. (blue) and Ly5.2 T cells (red).We could observe several NK cells in close proximity to naïve CD8 T cells, but only in mice infected with the virus containing the OVA epitope.
(4.23 MB TIF)
