# Supplementary figures and images for: Both Conventional and Interferon Killer Dendritic Cells Have Antigen-Presenting Capacity during Influenza Virus Infection
Source: PLoS One. 2009 Sep 28;4(9):e7187. doi: 10.1371/journal.pone.0007187 (PMC2747012; doi:10.1371/journal.pone.0007187)

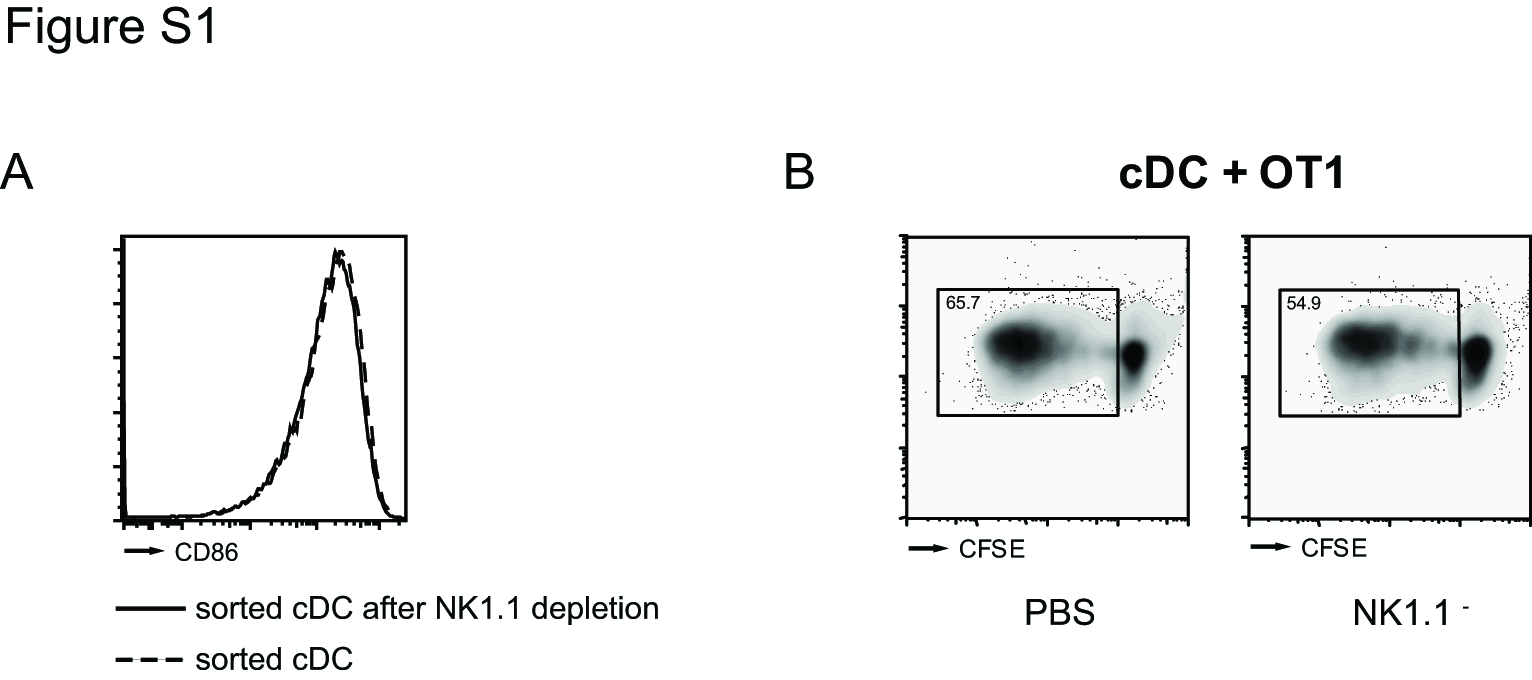

Supplement: Figure S1 — Effect of NK1.1 depletion of APC capacities of lung cDCs. (A) Expression of CD86 on cDCs taken at 4dpi from the lungs of influenza infected mice, that were treated with depleting NK1.1 antibody or isotype control. (B) Antigen presenting capacity of cDCs sorted from lung tissue at 4 days after infection with WSN containing an OVA-CD8+ epitope. Sorted cells were co-cultured for 4 days with CFSE-labelled OVA specific CD8 T cells (OT1). A comparison was made between cDCs sorted from NK depleted mice (NK1.1-) versus non-depleted mice, which received an isotype control injection (PBS). Numbers in top left corners represent the percentage of cells recruited into cell division. (4.77 MB TIF) [file pone.0007187.s001.tif]

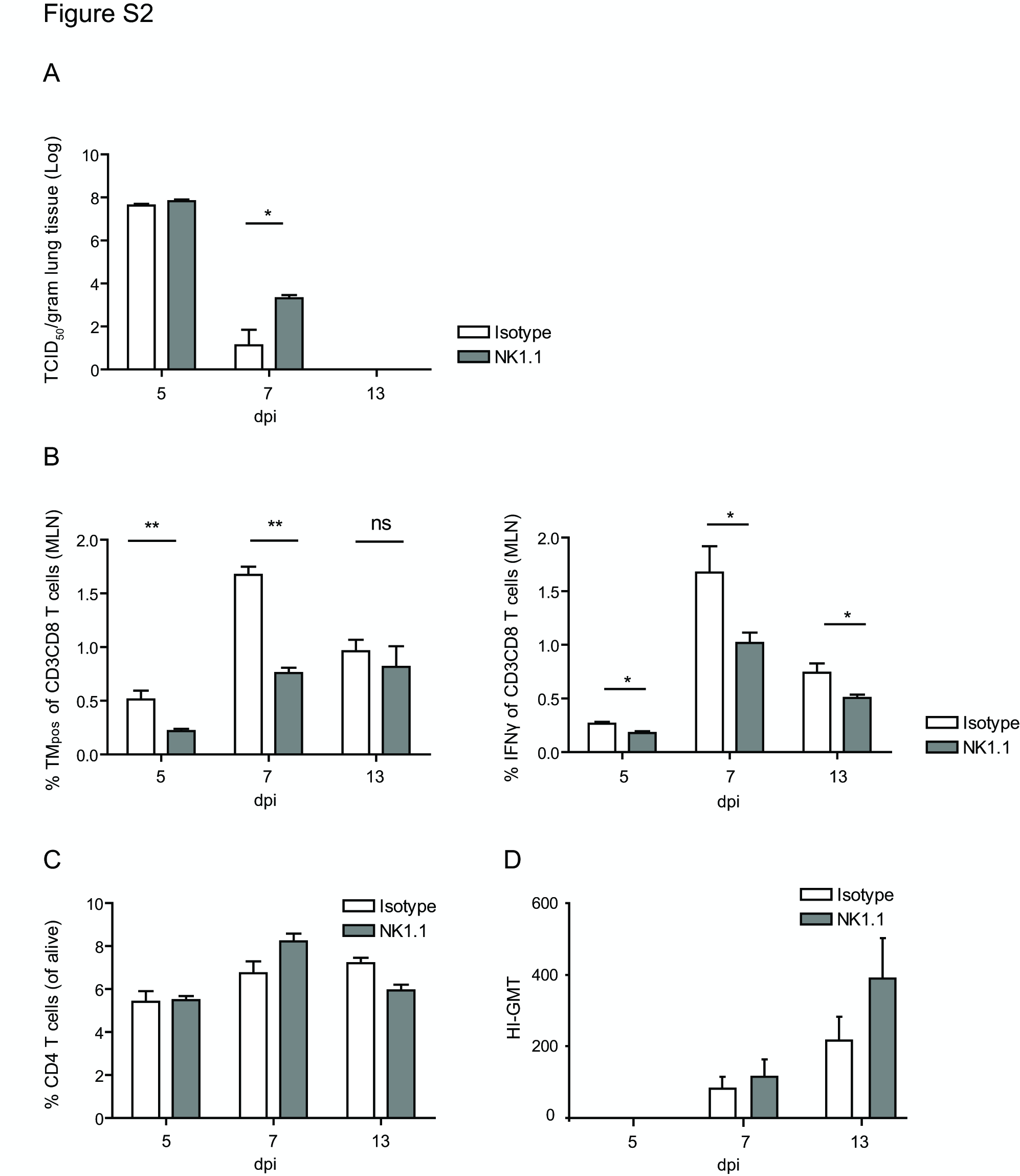

Supplement: Figure S2 — Depletion of NK1.1+ cells during in vivo influenza virus infection affects infection parameters at various time points following infection. Mice were treated with depleting NK1.1 antibody or isotype control during influenza virus infection. (A) Viral titers were measured in lung tissue at day 5, 7, and 13 post infection. (B) Left plot shows TM specific CD8+ T cells measured in MLN at day 5, 7, and 13 pos infection. Right plot demonstrates IFNγ producing CD8+ T cells in MLN, measured by intra-cellular staining. (C) % of CD4 T cells in MLN following infection. (D) Hemagluttinin specific antibodies in serum were measured at 8 dpi and depicted as geometric mean titer±SE. In all experiments at least 5 mice per group were used and values are expressed as mean±SEM. * p<0,05. (18.94 MB TIF) [file pone.0007187.s002.tif]

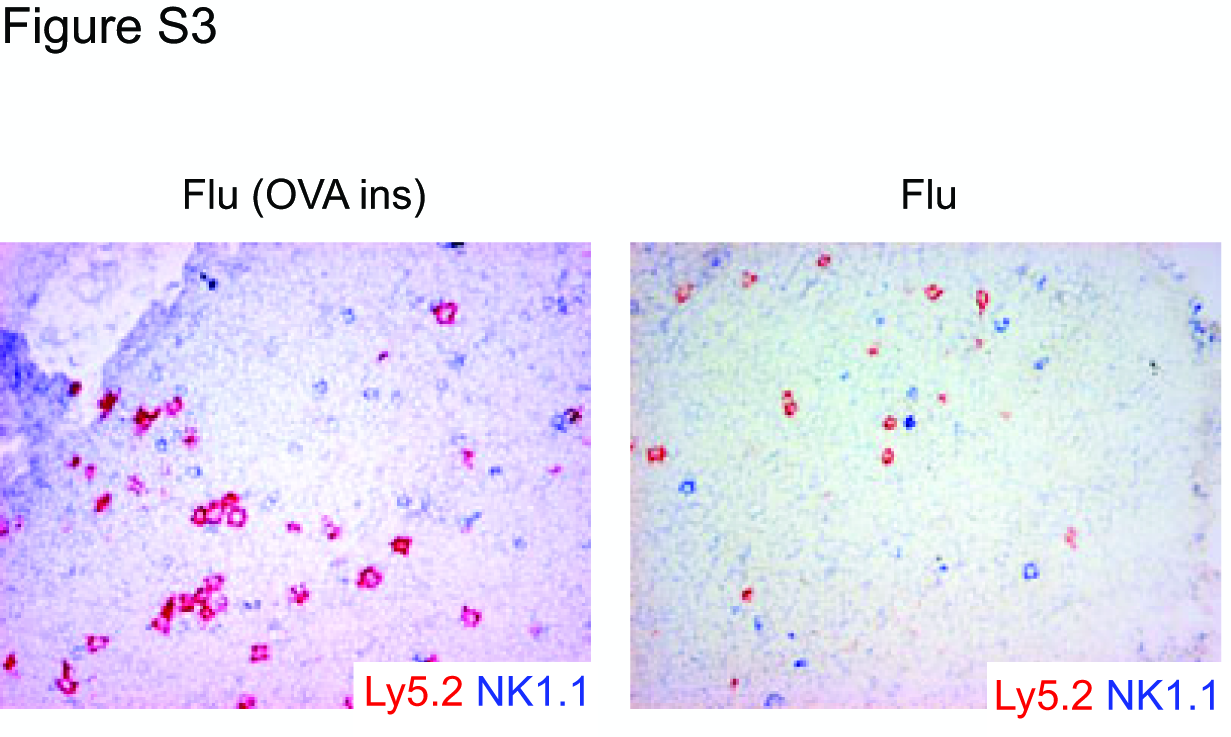

Supplement: Figure S3 — Colocalization of NK cells and naïve CD8 T cells in MLN at early time points post infection. Adoptive transfer of naïve Ly5.2 OTI CD8+ cells (reactive to OVA class I epitope) was performed to Ly5.1 recipients, allowing to detect OVA specific CD8 T cells using a Ly5.2 antibody. 1 day later mice were immunized with influenza virus containing the OVA class I epitope (Flu (OVA insert)) or with wild type influenza virus (Flu). At 2 days post infection MLN were stained for NK cells with an antibody against NK1.1. (blue) and Ly5.2 T cells (red).We could observe several NK cells in close proximity to naïve CD8 T cells, but only in mice infected with the virus containing the OVA epitope. (4.23 MB TIF) [file pone.0007187.s003.tif]
